# The Science of Learning Health Systems: Scoping Review of Empirical Research

**DOI:** 10.2196/34907

**Published:** 2022-02-23

**Authors:** Louise A Ellis, Mitchell Sarkies, Kate Churruca, Genevieve Dammery, Isabelle Meulenbroeks, Carolynn L Smith, Chiara Pomare, Zeyad Mahmoud, Yvonne Zurynski, Jeffrey Braithwaite

**Affiliations:** 1 Australian Institute of Health Innovation Macquarie University Sydney Australia

**Keywords:** learning health systems, learning health care systems, implementation science, evaluation, health system, health care system, empirical research, medical informatics, review

## Abstract

**Background:**

The development and adoption of a learning health system (LHS) has been proposed as a means to address key challenges facing current and future health care systems. The first review of the LHS literature was conducted 5 years ago, identifying only a small number of published papers that had empirically examined the implementation or testing of an LHS. It is timely to look more closely at the published empirical research and to ask the question, Where are we now? 5 years on from that early LHS review.

**Objective:**

This study performed a scoping review of empirical research within the LHS domain. Taking an “implementation science” lens, the review aims to map out the empirical research that has been conducted to date, identify limitations, and identify future directions for the field.

**Methods:**

Two academic databases (PubMed and Scopus) were searched using the terms “learning health* system*” for papers published between January 1, 2016, to January 31, 2021, that had an explicit empirical focus on LHSs. Study information was extracted relevant to the review objective, including each study’s publication details; primary concern or focus; context; design; data type; implementation framework, model, or theory used; and implementation determinants or outcomes examined.

**Results:**

A total of 76 studies were included in this review. Over two-thirds of the studies were concerned with implementing a particular program, system, or platform (53/76, 69.7%) designed to contribute to achieving an LHS. Most of these studies focused on a particular clinical context or patient population (37/53, 69.8%), with far fewer studies focusing on whole hospital systems (4/53, 7.5%) or on other broad health care systems encompassing multiple facilities (12/53, 22.6%). Over two-thirds of the program-specific studies utilized quantitative methods (37/53, 69.8%), with a smaller number utilizing qualitative methods (10/53, 18.9%) or mixed-methods designs (6/53, 11.3%). The remaining 23 studies were classified into 1 of 3 key areas: ethics, policies, and governance (10/76, 13.2%); stakeholder perspectives of LHSs (5/76, 6.6%); or LHS-specific research strategies and tools (8/76, 10.5%). Overall, relatively few studies were identified that incorporated an implementation science framework.

**Conclusions:**

Although there has been considerable growth in empirical applications of LHSs within the past 5 years, paralleling the recent emergence of LHS-specific research strategies and tools, there are few high-quality studies. Comprehensive reporting of implementation and evaluation efforts is an important step to moving the LHS field forward. In particular, the routine use of implementation determinant and outcome frameworks will improve the assessment and reporting of barriers, enablers, and implementation outcomes in this field and will enable comparison and identification of trends across studies.

## Introduction

### Background

Contemporary health care systems are not always fit for purpose or evidence-based [1,2]. Despite all the resourcefulness and efforts internationally, health care performance has, by and large, flatlined, with persisting iatrogenic harm, inefficiencies, and health care waste [2,3]. To overcome ongoing challenges in health care systems, there is growing awareness of the need for health care systems predicated on knowledge harvesting and exploitation, and continuing improvement through leveraging big data and incorporating patients’ perspectives and choices into decisions [2,4]. The concept of a learning health system (LHS) was first formally discussed at a Roundtable on Evidence-Based Medicine in 2007 [5]. There is now widespread recognition that what is needed is a health care system that “consistently delivers reliable performance and constantly improves, systematically and seamlessly, with each care experience—in short, a system with an ability *to learn*” [6].

### The Vision for, and Progress Toward, an LHS

An LHS has been described by the US Institute of Medicine (IoM; now the National Academy of Medicine) as one where science, informatics, incentives, and culture are aligned for enduring continuous improvement and innovation; best practices are seamlessly embedded in the care process; patients and families are active participants in all elements; and new knowledge is captured as an integral by-product of the care experience [7]. Priorities for achieving this lofty, aspirational vision include advancing the development of a fully interoperable digital infrastructure, the application of data-driven research within health care, and a culture of transparency on outcomes and cost [8]. Although this vision has remained largely aspirational to date, rapid innovations in big data, machine learning, and artificial intelligence (AI) are creating the opportunity, and expectation, that health care systems can make real the promise of an LHS [4,9,10]. For example, in the United States, well-regarded health care provider Geisinger reported on its significantly expanded informatics and science capabilities over the past 5 years by migrating its comprehensive data assets into a big data enterprise data warehouse infrastructure [11]. Geisinger documented its efforts to improve patient-clinician engagement with patient-reported experience measures (PREMS) serving as the primary metric for measuring success, moving Geisinger into closer alignment with the LHS vision [11].

### Empiricizing LHSs

**Figure 1:**
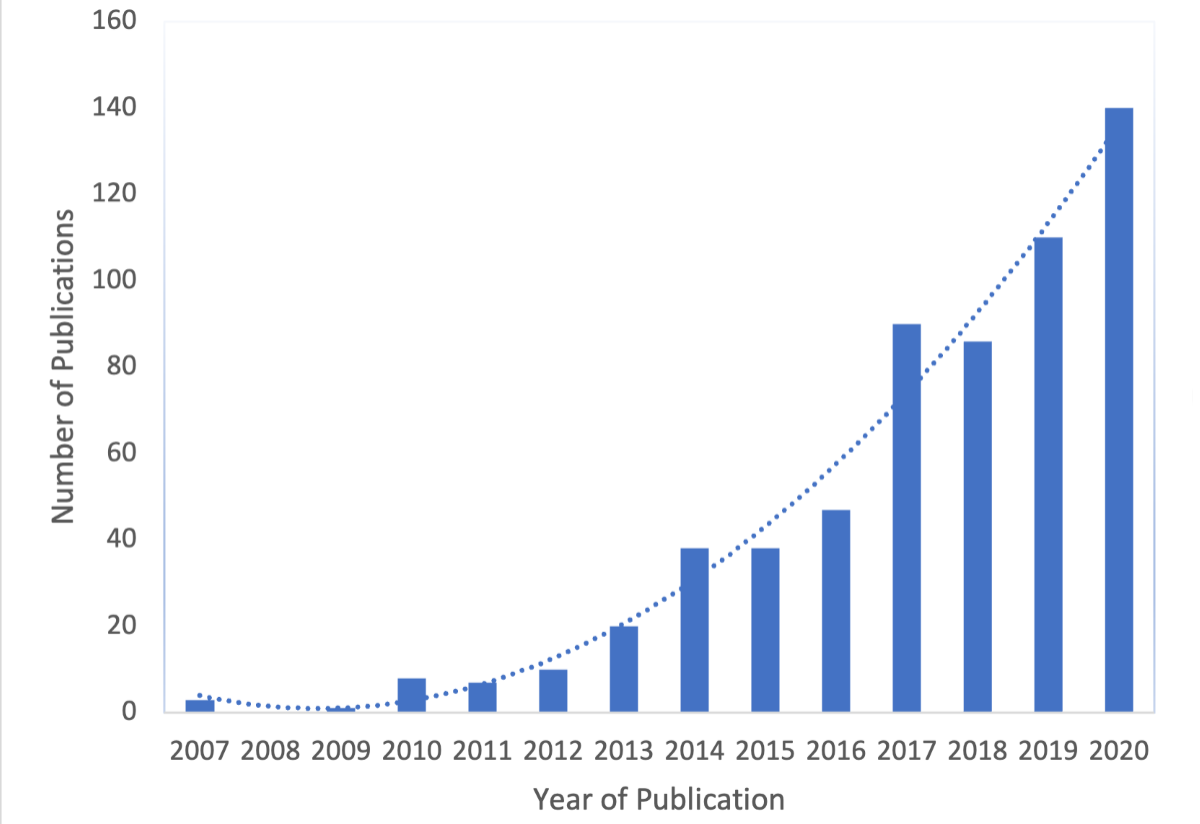
Increase in publications on LHS over time, 2007-2020 (generated using data from PubMed on publications returned using the search term “learning health system” OR “learning health care system”). LHS: learning health system.

Despite enthusiasm for big data and AI as the learning cornerstones, the question remains whether there is compelling evidence for the successful implementation of programs, systems, and services that are making marked progress toward approximating the normative descriptions of the LHS. Research interest in LHS concepts and ideas has been increasing, as evidenced by the growing number of publications on LHS since it was first discussed in 2007 (Figure 1) and the emergence of the influential journal *Learning Health Systems* [12]. Several reviews of the topic have also now emerged, identifying limited but growing empirical LHS applications. In 2016, Budrionis and Bellika [13] conducted a systematic review of the LHS literature, revealing that of the 32 identified papers, only 13 (40.6%) empirically examined the implementation or testing of an LHS. They also found that of the empirical evaluations, most suffered from substantial methodological and data limitations. Two years later, in 2018, Platt et al [14] conducted a scoping review, showing that although most of the research was theoretical, there was a growing number of empirical publications within the LHS domain [14]. More recently, Enticott et al [15] identified 23 LHS environments internationally; most were enabled by digital data gathered by electronic health records. However, these initiatives were largely identified from gray-literature sources (reports and policies) that were not designed as robust studies to create quality research evidence [15].

With the growth in empirical contributions in the LHS field, it is timely to examine the published empirical research and to determine the status of the field, 5 years on from the first LHS review of Budrionis and Bellika [13]. For this review, we defined an empirical study as one that reports primary or secondary data gathered by means of a specific methodological approach [16]. We seek to leverage recent developments from the field of implementation science, which aligns closely with a core goal of LHSs, to get more evidence into practice, and to satisfy requirements for continuous quality improvement [17-19].

### This Study

In this paper, we report on a scoping review of empirical research within the LHS domain. We map out the empirical research that has been conducted to date, identify limitations, and identify future directions for the field. The scoping review was designed to answer questions in 3 key research areas:

- What types of empirical contributions within the LHS domain have been conducted?
- What have been the key areas of research?
- What study designs and research methods have been used?

Among the empirical studies examining implementation:

- What implementation outcomes have been examined and what implementation determinants have been identified?
- Which implementation science frameworks and tools have been used?
- What are the current knowledge gaps and methodological limitations of empirical research in the LHS field?

## Methods

### Study Design

Our scoping review followed a protocol that was developed in accordance with the Preferred Reporting Items of Systematic Review and Meta-Analyses Extension for Scoping Reviews (PRISMA-ScR) [20]. A scoping review method, which examines the extent, range, and nature of empirical work on a topic, was used to identify gaps and provide suggestions to improve future empirical research on LHSs [21]. For this review, which focused further on the implementation of an LHS, implementation determinants were defined as barriers and enablers that may prevent or facilitate, respectively, improvements in practice [22], as reported in the included studies. The implementation outcomes taxonomy by Proctor et al [23] was used as a systematic framework for examining implementation-focused LHS studies (ie, acceptability, adoption, appropriateness, feasibility, fidelity, implementation cost, penetration, and sustainability), distinguishing these from service and patient outcomes.

### Search Strategy

A search strategy was developed by the research team and executed in January 2021. Two academic databases (PubMed and Scopus) were searched from January 1, 2016, to January 31, 2021, using the term “learning health* system*”.

### Inclusion and Exclusion Criteria

Papers were included if they were (1) published from January 1, 2016, to January 31, 2021, (2) had an explicit focus on LHSs, and (3) were empirical studies. Studies reporting primary or secondary data were considered empirical so long as they provided sufficient information about their methodological approach [16,24]. Peer-reviewed journal articles, peer-reviewed full conferences papers, and book chapters that provided sufficient information about their methodological approach and results were also included. Study protocols, review papers, journal commentaries, and editorials were excluded. Studies not in the English language and not explicitly about LHSs (eg, only used the term in the abstract or conclusion) were also excluded.

### Eligibility Screening

Reference details (including abstracts) were downloaded into the reference management software Endnote X9 [25]. The review team (authors LAE, MS, CP, ZM, and IM) screened the full-text publications to determine their inclusion against criteria, and 5% of the retrieved publications were independently screened by the entire review team to ensure consistent inclusion. Any discrepancies among reviewers’ judgements were reviewed by 2 authors (LAE and MS) in consultation with authors YZ and JB.

### Data Extraction

Relevant information was extracted at the full-text review stage using a purpose-designed workbook in Microsoft Excel 365 and included (1) publication details (paper title, year, country of residence of corresponding author, paper type, and paper keywords); (2) primary study focus (thematically coded after data extraction); (3) study context (clinical, hospital, health care system); (4) study design (quantitative, qualitative, mixed methods); (5) study data type (primary or secondary); (6) implementation framework, model, or theory used; and (7) implementation determinants or outcomes examined.

### Assessment of Evidence Quality

Consistent with the LHS review by Enticott et al [26], the Grading of Recommendations Assessment, Development and Evaluation (GRADE) approach was applied to assess the overall quality of evidence based on the study design [26]. Using the GRADE approach, randomized trials without important limitations provide high-quality evidence, while observational studies without special strengths (eg, the use of an implementation science framework) or with important limitations provide low-quality evidence. GRADE recommends that design factors, such as concurrent controls, can improve the quality of evidence; therefore, studies with concurrent controls without important limitations were assessed as providing medium-quality evidence.

### Data Synthesis and Analysis

Papers were grouped together based on extracted data (eg, study design) and summarized through narrative techniques. The country of the corresponding author was coded by income classification based on World Bank definitions of the gross national income per capita. The 3 categories were low (<US $1045), middle (US $1046–$12,695), and high (>US $12,696) income [27].

Overarching topic areas were identified through an inductive analysis of publication keywords by 2 authors (LAE and CP). These were extracted by the research team and then cleaned and checked for consistency. During data cleaning, keywords were consolidated in the case of plurals (eg, “intervention” vs “interventions”); however, keywords were kept independent in the case of arguably consistent meaning but different phrasing (eg, “learning health care system” vs “learning health system”) in order to represent the variability of terms used in the LHS field. The keyword data was analyzed for frequency and co-occurrence and graphically presented using Gephi version 0.9.2.

The primary study concern or focus was inductively classified by 2 authors (LAE and MS) into 1 of 4 classifications: (1) specific programs, systems, and platforms; (2) ethics, policies, and governance; (3) stakeholder perspectives of LHSs; and (4) LHS-specific research strategies and tools. Studies that examined implementation outcomes were further reviewed and classified by the 2 authors (LAE and MS) according to 8 implementation outcome categories [23], distinguishing these from service and client outcomes, and with definitions tailored to suit the LHS context (Table 1).

**Table 1:** Definition of implementation outcomes [23].

Domain	Definition
Adoption	Uptake of the LHS^a^ initiative by health care professionals and health services
Acceptability	Health care professionals’ satisfaction with various aspects of the LHS initiative (eg, content, complexity, comfort, delivery, and credibility)
Appropriateness	Fit, relevance, compatibility, suitability, usefulness, and practicability perceived by health care professionals and patients
Feasibility	The actual fit, utility, and practicability of the program within a health service setting and its subsystems, as reported by health care professionals and managers
Fidelity	The LHS initiative delivered, as intended; adherence by health care professionals; and quality of program delivery
Cost	Financial impact of LHS implementation to the health service or organization
Penetration	Spread or reach of the LHS initiative assessed at the organization or setting level
Sustainability	The extent to which the LHS program is maintained or institutionalized within a health service’s standard operations

^a^LHS: learning health system.

## Results

### Description of Included Studies

**Figure 2:**
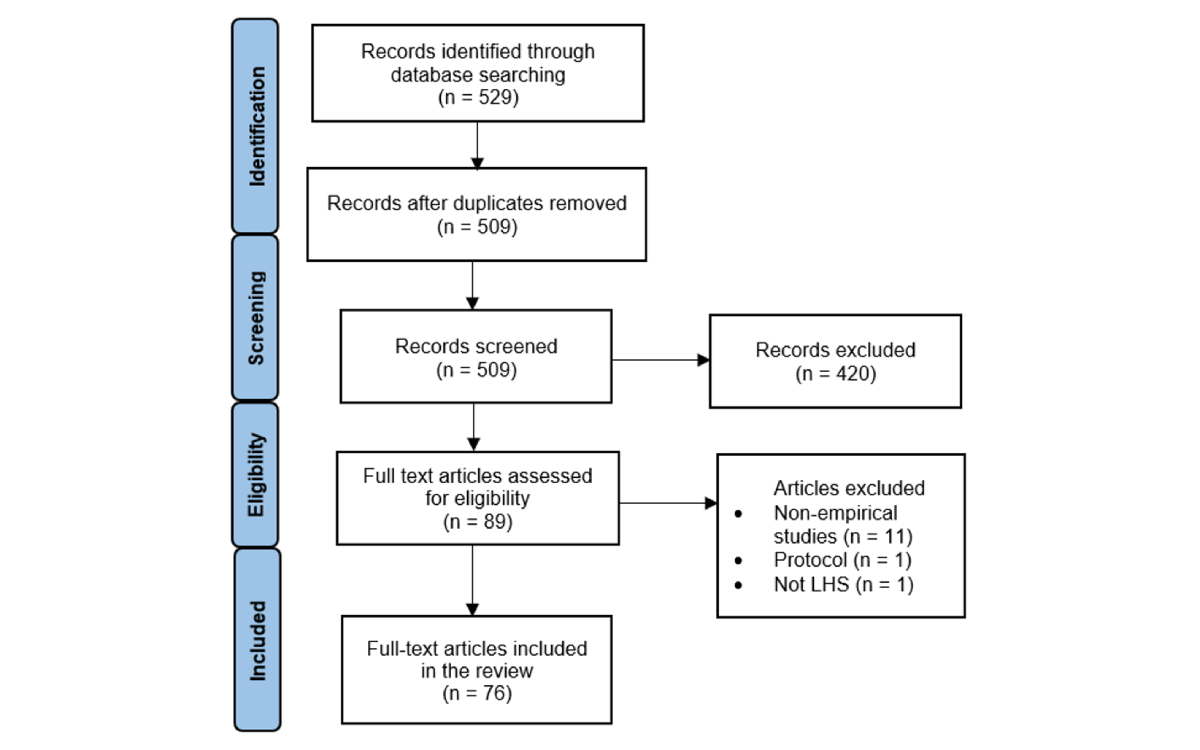
Search and review strategy. LHS: learning health system.

The search identified a total of 529 citations. After removing duplicates, 509 (96.2%) remained for title/abstract review. During the title/abstract screening, 420 (82.5%) studies were discarded as not meeting the inclusion criteria. Based on the full-text assessment, a further 13 (14.6%) of 89 studies did not meet the inclusion criteria, and hence 76 (85.4%) studies were included in this review (Figure 2).

A summary of the key characteristics of the included studies is presented in Table 2 (also see Multimedia Appendix 1 for details of all included studies). Of the 76 included studies, the majority (n=72, 94.8%) were published in peer-reviewed journals, 3 (3.9%) were full conference papers, and 1 (1.3%) was a book chapter. The 72 papers were spread widely across 54 different journals, with *Learning Health Systems* (n=7, 9.7%) and *eGEMS* (*Generating Evidence & Methods to Improve Patient Outcomes*; n=4, 5.6%) being the most popular.

**Table 2:** Summary of key characteristics of the included publications (N=76).

Classification		Papers, n (%)
**Country of corresponding author**		
	United States	55 (72.4)
	United Kingdom	9 (11.8)
	Canada	2 (7.6)
	France	2 (2.6)
	Germany	2 (2.6)
	The Netherlands	2 (2.6)
	Other	4 (5.3)
**Country income classification**		
	High	75 (98.7)
	Middle	1 (1.3)
	Low	0
**Study methods**		
	Quantitative methods	42 (55.3)
	Qualitative methods	27 (35.5)
	Mixed methods	7 (9.2)
**Study data type**		
	Primary data	46 (60.5)
	Secondary data	23 (30.3)
	Both primary and secondary data	7 (9.2)

The location of studies was predominantly restricted to high-income countries, with most coming from the United States (n=55, 72.4%), followed by the United Kingdom (n=9, 11.8%), and Canada (n=3, 3.9%). Over half of the studies (n=42, 55.3%) were quantitative studies, around one-third (n=27, 35.5%) were qualitative and the remaining (n=7, 9.2%) were mixed-methods studies. Although most studies (n=46, 60.5%) utilized primary data alone, one-third of the studies (n=23, 30.3%) relied on secondary data sets, such as electronic health records and data repositories, and a smaller number (n=7, 9.2%) used both primary and secondary data sets.

**Figure 3:**
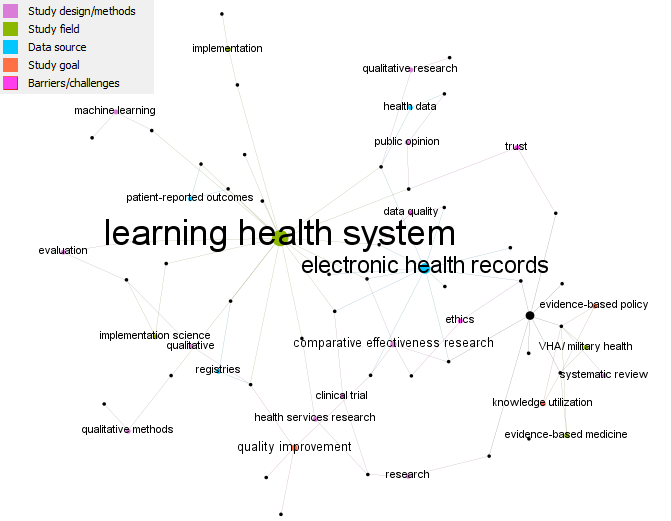
Network of co-occurring keywords with in-degree ≥2. Each circle (node) is a keyword, and each line (edge) represents co-occurrence. The size of the node indicates the number of times a keyword was used. Colors represent different topic areas. VHA: Veterans Health Administration.

Of the 76 included studies, 52 (68.4%) listed keywords, with 190 keywords in total. The most common keywords (ie, those with the highest in-degree score) were “learning health system” (n=20), “electronic health records” (n=11), and “leaning health care system” (n=8). A network of keywords was created to demonstrate keywords frequently used in papers together; Figure 3 visually summarizes overarching topic areas of the empirical papers on LHSs identified in this review. To aid interpretation, only keywords with in-degree scores (ie, number of ties directed to or received by a node) greater than or equal to 2 are displayed (see Figure 3). The size of the node is indicative of frequency (larger nodes indicate a higher number of papers using the keyword). The line between 2 nodes (tie) indicates keywords used together in a paper. Our inductive categorization of keywords identified 4 broad topic areas into which these words fell: (1) study design/methods (eg, comparative effectiveness research, clinical trial, qualitative research), (2) study field (eg, health services research, implementation science), (3) data source (eg, electronic health records), (4) study goal (eg, quality improvement), and (5) barriers/challenges (eg, ethics, data quality). In Figure 3, these are colored separately to indicate keyword categories in relation to one another.

The thematic analysis of the study focus led to classification into either (1) specific programs, systems, and platforms or 1 of the following key research areas: (2) ethics, policies, and governance; (3) stakeholder perspectives of LHSs; or (4) LHS-specific research strategies and tools. This classification system was used to break down studies and separately synthesize information on the study field, setting, population, and study design, as well as implementation determinants and outcomes examined. These categories of focus are considered separately later.

### LHS Programs, Systems, and Platforms

#### Implementation and Validation Issues

Over two-thirds of the included studies (53/76, 69.7%) were concerned with implementing a particular program, system, or platform designed to contribute to achieving an LHS. For example, Bhandari et al [28] described the application of a national health outcomes information registry for pain medicine that had been adapted to pediatric populations, reporting on the technical, financial, and systems considerations of using retrospective data. Of these 53 program-specific studies, 37 (69.8%) focused on a particular clinical context or patient population; most commonly oncology (n=7, 18.9%), neurology (n=4, 10.8%), and pediatrics (n=4, 10.8%). The remaining 16 studies (30.2%) focused on whole hospital systems (n=4, 25%) or on other broad health care systems encompassing multiple facilities (n=12, 75%), such as the US Veterans Health Administration (VHA). In over half of the studies (27/53, 50.9%), the implemented LHS involved examination and use of data from electronic health records, clinical registries, or other routinely collected data sources. Most of the program-specific studies (37/53, 69.8%) utilized quantitative methods, with a smaller number utilizing qualitative methods (10/53, 18.9%) or mixed-methods designs (6/53, 11.3%).

In addition, 9 (16.9%) of 53 studies reported on the validation of a specific LHS program or system. These studies sought to develop the data infrastructure and predictive tools to enable the realization of an LHS within specific care contexts or across entire health care systems. One such study by Ethier et al [29] sought to validate the embedding of clinical trial functionalities into routine electronic health record systems that could then form part of an LHS in European primary health care services. Although their approach allowed precise prospective mapping of data elements within electronic health records, the authors found that patient-related outcome measures (PROMs) are less often completed electronically than they are in paper form. The authors emphasized that future efforts may need to focus on optimizing the delivery of PROMs within LHSs.

#### Barriers, Enablers, and Outcomes

Almost one-third (16/53, 30.2%) of program-specific studies considered the barriers and enablers to the implementation of specific programs, systems, or platforms. This progression from the predominantly theoretical contributions to the LHS literature to more applied and empirical evaluations has begun to uncover the potential methodological flaws and limitations of data systems in realizing the promise of an LHS. In 1 study of a US multicenter research program embedded within the VHA system, a survey of LHS decision makers who accessed the VHA Evidence Synthesis Program (ESP) identified that that the ESP information and reports are most frequently used to develop clinical guidance, identify future research needs, and determine implementation strategies, particularly surrounding adoption decisions and medical device procurement [30]. In another study, the use of web-based platforms and tools was identified as necessary but not sufficient in themselves to realize an LHS [31]. For example, clinicians often reject decision support system recommendations when patients present with complex comorbidities that might not be adequately considered by the system [32]. When implementing LHSs, stakeholder engagement to identify data-driven solutions to improve health care was considered feasible but resource intensive [33].

There were many barriers to the implementation of LHS systems. A lack of relevant evidence and information about how to translate research findings in practice presented a key challenge to applying the concept of an LHS in reality [34]. Change resistance, resource constraints, and concerns regarding centralized decision making were prominent barriers to the ability to transform care delivery [34,35]. Political pressures to implement therapies or technologies with uncertain or little evidence [34], technical challenges and implications for security of patient data [36,37], practical constraints in reconfiguring clinician-patient relationships [36,38], and the ability to meet patient expectations and satisfaction regarding care [39] were also frequently reported barriers. Important enablers included the timely provision of clear data that are understood, trusted, and clinically useful [34,36,40]; facilitation of clinician willingness to volunteer data [41]; and flexible systems that are embedded within electronic health records and support engagement with data as part of the normal clinical workflow and joint decision making [34,36,40,42]. Social conditions that promote clinicians and patients to work together and minimize barriers to patient participation [36,43], promoting respect, trust, relationships, collaboration, and communication among clinicians [44], and constructive and nonpunitive approaches to providing feedback and reducing errors [45] also represented prominent solutions to overcome identified barriers.

**Figure 4:**
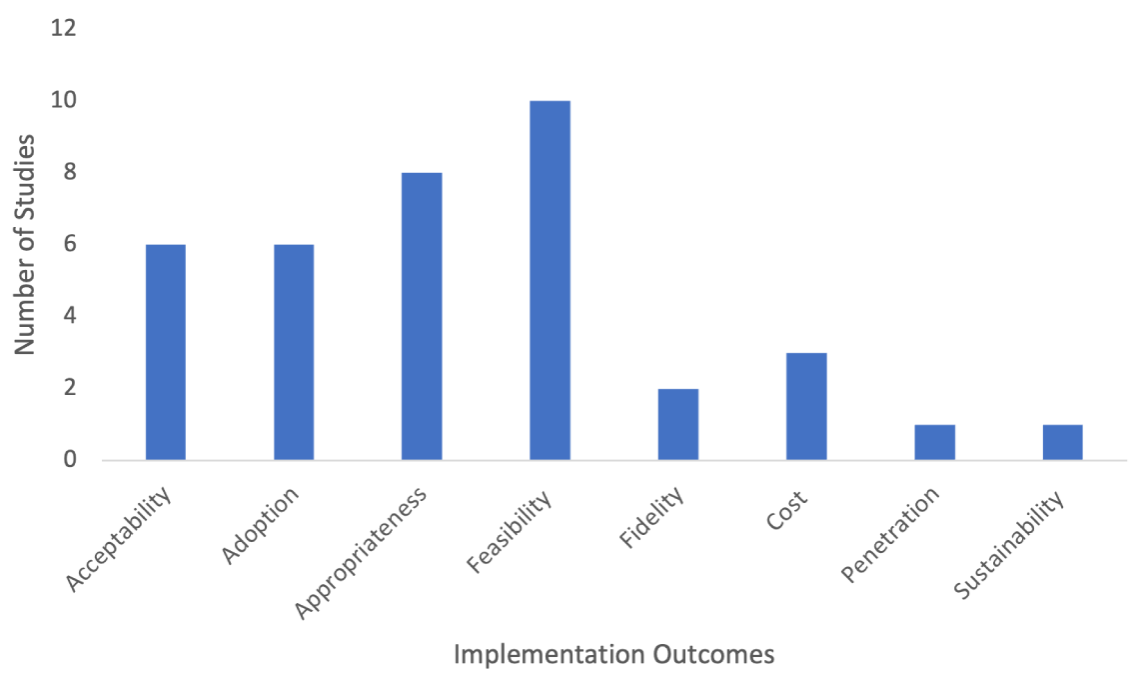
Number of studies reporting by implementation outcome.

Of the 53 LHS program-specific studies, 16 (30.2%) were classified as assessing outcomes according to the Proctor implementation outcomes taxonomy [23] (Figure 4). Most assessed feasibility (8/16, 50%) [28,46-54], appropriateness (7/16, 43.8%) [28,41,44-46,52,54,55], acceptability (6/16, 37.5%) [28,44-46,52,56], and adoption (6/16, 37.5%) [28,35,45,46,57,58]. Less commonly studied implementation outcomes were implementation cost (3/16, 18.8%) [28,47,48], fidelity (2/16, 12.5%) [28,46], sustainability (1/16, 6.3%) [35], and penetration (1/16, 6.3%) [46]. This emphasis on the outcomes that are salient at earlier stages of implementation, such as the feasibility, appropriateness, and acceptability of an LHS, highlights the burgeoning nature of the field, with few LHSs having progressed to questions around sustainability, penetration, and fidelity.

Of the 53 LHS program-specific studies, only 1 (1.9%) structured its evaluation using an implementation science framework [35]. This mixed-methods study sought to evaluate the VHA Innovation Ecosystem, which includes the Diffusion of Excellence (DoE) program that identifies and diffuses gold status practices across VHA facilities. In this study, the Consolidated Framework for Implementation Research (CFIR) [59] was used to inform qualitative data collection and direct content analysis for the identification of barriers and enablers influencing implementation and affecting sustainability [35].

### Research Area: Ethics, Policy, and Governance

For 10 (13.2%) of 76 studies, ethics, policy, or governance was the primary focus. These studies examined LHS ethics, policy, and governance issues through qualitative interviews (n=3, 30%) [60-62] or focus groups (n=3, 30%) [63-65], quantitative methods (n=3, 30%) [66-68], or mixed-method designs (n=1, 10%) [69]. Participants in these studies included health care consumers [63,66-69], ethical review board members [61,64], institutional leaders [62], health care providers, managers, and researchers [60,61,65].

Although none of these studies examined implementation effectiveness, each study explored broad ethical, policy, or governance barriers and enablers to achieving an LHS. The implications of sharing data were a central concern in all 10 studies. Specific concerns regarding health data sharing included the patients’ right to consent to, and to be notified of, data sharing [66], patient privacy [63], and profit-driven data custodianship [69]. Studies found statistically significant factors influencing health consumers’ positive attitudes toward data sharing, including higher education, low concerns regarding privacy [67,68], and the belief that participation in research is an ethical imperative [68]. Societal altruism was also commonly discussed in qualitative studies [69]; focus group studies found that when educated on societal benefits of data sharing, health consumers were more likely to be amenable to it [63]. Other studies suggested that transparency and trust could improve health data-sharing concerns [60,65,68,69], and opt-out consent policies were an acceptable method of increasing participation in data sharing to support LHSs [63,66].

Research practices were raised as an issue in 5 (6.6%) of 76 studies [60-62,64,65]. Issues often stemmed from the ambiguity between what is classed as research, which is subject to ethical oversight, and consent, and transparency policies, and what is considered quality improvement, which is often exempt from such governance [62,64]. The divide raised ethical concerns, including the potential for studies to be inappropriately classed as quality improvement in order to expedite LHS feedback loops [64] and researchers undertaking more rigorous research practices, such as randomization or implementing randomization without consent [62,65]. To overcome this, studies suggested that the segregation between research and quality improvement was not appropriate and collective governance was recommended for all improvement practices [61] as were accelerated ethical processes [65].

### Research Area: Stakeholder Perspectives on LHS

Five (6.6%) of 76 studies examined stakeholders’ perspectives on particular components of an LHS, including quality improvement [70], electronic prescribing and medicines administration systems [71] and diagnostic practices [72]. The studies were all qualitative and used either interviews [72-74] or focus groups [70,71]. The participants in these studies were junior doctors [70], health system leaders [73], researchers [72], and other diverse health care system stakeholders [71,74]. Psek et al [73], for example, interviewed 41 senior leaders across an integrated health delivery system, identifying 10 themes related to operationalizing an LHS, such as “balancing learning and workflow” and “integrating cultural and operational silos.” Although not strictly implementation evaluation studies, all 5 studies under this category identified barriers and facilitators relevant to the realization of an LHS, including the usability of systems [71] and time constraints, such as time for participation in quality improvement activities [70].

### Research Areas: LHS-Specific Research Issues and Tools

Five (6.6%) of 76 studies described the novel development and application of LHS-specific research tools or frameworks [18,31,46,75,76], and 3 (60%) of these 5 studies outlined the development of rapid analytic tools to address the need for timely feedback and evaluation [18,46,76] and to address the limitations of traditional plan-do-study-act (PDSA) models [18]. For example, Brown-Johnson et al [18] outlined their qualitative approach and communication tool, the Stanford Lightning Report Method, which, using the coding structure of the CFIR, compared implementation evaluation barriers and enablers across 4 projects to explore the sensitivity of the method and the potential depth and breadth of the method findings. Their study suggested that the tool facilitates partnered qualitative evaluation and communication with stakeholders by providing real-time, actionable insights into dynamic health care implementation. In another study, Holdsworth et al [46] outlined an adapted rapid assessment procedure (RAP), which incorporates the Reach, Effectiveness, Adoption, Implementation and Maintenance (RE-AIM) framework and CFIR implementation science frameworks, and iterative working with stakeholders, as well as rapid team analysis and triangulation of data sources [46]. In this study, the authors presented case summaries of 4 academic medical centers to demonstrate the value of including RAPs in LHS research. This showed how contextually rich information can be produced using robust data collection methods within a short time frame. Two other studies outlined the development and application of implementation frameworks specifically for LHSs [31,75]. Safaeinili et al [75] conducted a qualitative study to develop an adapted version of the CFIR that would be more accessible and relevant for assessing barriers and enablers in the context of patient-centered care transformations within an LHS [75]. Franklin et al [31] developed an implementation framework to guide PROM data collection, interpretation, and use. The framework was designed with the aim of ensuring that future PROM implementation efforts across LHSs would capture PROMs at the correct time and, with associated risk factors, generate meaningful information to serve diverse stakeholders [31].

In addition, 3 (3.9%) of 76 studies examined LHS-specific research issues through the exploration of barriers and enablers to engaging participants, including clinicians and patients and carers, in research for health care organizations seeking to become LHSs [77-79]. For example, the study of Ciemins et al [78] surveyed 4 community-based health systems and found that although engaging clinicians in research is a step toward LHS attainment, infrastructure support and cultural commitment across the health care system are also required. They suggested that providing highly research-motivated clinicians with some dedicated research time might facilitate uptake [78]. Forrest et al [79] undertook interviews using a modified Delphi study to identify LHS researcher core competencies, with a total of 33 core competencies being prioritized around several domains. These included having complex systems knowledge, having expertise in implementation science and informatics, knowing when and how to use mixed-methods designs, and ensuring the engagement of all relevant stakeholders (eg, patients, clinicians) [79].

### Quality Assessment

The GRADE level of evidence for the included studies is provided in Multimedia Appendix 1. The level of evidence was assessed as high for 2 (2.6%) of the 76 studies that incorporated randomized controlled trial designs [53,80]. The level of evidence was rated medium for 11 (14.5%) quantitative studies with case comparisons or controls and 4 (5.3%) cross-sectional studies with special strengths because they incorporated implementation science frameworks within the design and analysis phases. A low level of evidence was assigned to 59 (77.6%) studies reporting observational data from registries, electronic medical records, or qualitative interviews without special strengths.

## Discussion

### Principal Findings

Since the 2016 review by Budrionis and Bellika [13], which found only 13 LHS empirical studies from 2007 to 2015, we identified a further 76, showing the growth of empirical applications within the LHS field over the past 5 years. Almost three-quarters (n=55, 72.4%) of the studies were from the United States, and virtually all (n=75, 98.7%) were from high-income countries. Over half of the studies (n=42, 55.3%) were quantitative, with just over one-third (n=27, 35.5%) being qualitative studies and a smaller proportion (n=7, 9.2%) being mixed-methods studies. Progress is clearly being made in empiricizing the LHS in differing settings and jurisdictions.

Each of these studies was classified into an area of primary focus, with over two-thirds of them being concerned with implementing a particular program, system, or platform designed to contribute to achieving an LHS. Most of these studies examined data from electronic medical records or registries, aligning with the findings from our keyword analysis, and from recent research [15]. Most of these studies also focused on a specific clinical context or patient population, potentially explaining why the papers were widely spread across different journals. Few studies focused on whole hospital systems or on other broad health systems encompassing multiple facilities, suggesting that research into LHSs remains locally focused and in specific clinical care contexts. These results align with recommendations on decision making around project scale, with some emphasizing the importance of demonstrating the effective implementation of an LHS at a smaller scale first, which would then arguably provide the motivation and resources for a large-scale implementation to follow [13]. Large-scale LHS implementation efforts can also be slowed down by challenges arising from system and contextual complexities [13].

The number of studies focused on implementing LHSs is increasing. This raises the meta-question, Have the benefits of an LHS been empirically demonstrated prior to implementation? LHS research is a radically applied field of inquiry that lends itself well to real-world evaluations, utilizing natural experiments in situ [81,82]. By leveraging study designs that evaluate the effectiveness of LHS-specific programs, systems, and platforms simultaneously with their implementation, there is an opportunity to accelerate the generation of empirical evidence for LHSs. For example, effectiveness-implementation hybrid studies are increasingly being applied in implementation science, and these provide an appropriate design for the study of LHSs, where interventions tend to be complex and where multiple interrelated factors need to be considered to ensure implementation is both sustained and effective [83].

Few of the implementation-focused studies included in this LHS review framed their evaluations using an implementation framework or reported on implementation outcomes. Although there is a plethora of implementation science theories, models, and frameworks available [22], their use in LHS research remains limited. The incorporation of implementation science frameworks can provide a structured and pragmatic approach to plan, implement, and evaluate interventions. The CFIR [59] is 1 of the most widely used determinant frameworks, designed specifically to systematically assess barriers and facilitators to implementation within local settings, that can help guide decisions about the needs of the local context [84]. In contrast, the Proctor taxonomy of implementation outcomes [23] and RE-AIM [85] are examples of implementation science frameworks that can be applied to evaluate implementation [22]. Other frameworks for implementing and assessing telemedicine applications, such as the Model for Assessment of Telemedicine (MAST), have also been suggested as having potential applicability in understanding and evaluating the implementation of LHS programs, systems, and platforms [13]. The field of LHSs would benefit from the systematic and integrated use of frameworks such as these, not just for the initial planning and summative evaluation, but also to evaluate interim progress, ensure the suppression of unintended consequences, and help guide appropriate adaptations [86].

In the relatively small number of included studies where implementation outcomes were measured, studies tended to focus on outcomes related to the early stages of implementation, assessing the feasibility [28,47-54], appropriateness [28,41,44-46,52,54,55], acceptability [28,44-46,52,56], and adoption [28,35,45,46,57,58]. This likely reflects that LHSs remain a relatively new service model that has not been widely implemented in a cohesive way over the longer term to be concerned with assessing the sustainability and penetration of LHS programs, systems, and platforms. Nevertheless, many studies are beginning to illustrate the barriers and enablers to implementing LHSs across different settings, which can inform future efforts to overcome resistance to progress or other challenges. Even included studies that did not explicitly focus on implementation identified system barriers relating to ethics, policy, and governance, with issues associated with data sharing featuring most prominently [60-69]. Stakeholder perspectives on system barriers were also identified, including the usability of systems and time constraints working in an LHS [71]. Understanding these barriers and enablers is a key first step toward unlocking the mechanisms that could trigger lasting improvements in how health care is delivered [87].

It is promising that we are also beginning to see the development of LHS-specific research tools. Traditional PDSA models, utilized to address the need for timely feedback within an LHS, have almost exclusively focused on quantitative patient data or process metrics [18]. Although PDSA cycles may be useful to identify whether an approach or intervention is effective, more timely feedback is needed to inform *how* and *why* an intervention is successful or unsuccessful [18]. Mixed-methods studies, including the incorporation of quantitative data from secondary sources and primary qualitative data, incorporate a more robust design for the LHS field, which has traditionally lacked mixed-methods approaches [46]. The use of quantitative data alone does not produce the depth of understanding of barriers and enablers to innovation, implementation, and measurement, nor does it generate lessons with the level of granularity needed to interpret the findings across a complex LHS [46]. Although qualitative data analysis methods are traditionally labor intensive, new qualitative approaches are emerging that include rapid qualitative data analysis [18,46] and the use of tailored implementation science frameworks for applicability in the context of patient-centered health care interventions [75] and for guiding future PROM implementation efforts across LHSs [31]. Although we identified relatively few studies incorporating an implementation science framework, we expect to see that application of such frameworks, and also tailored frameworks, will grow in the coming years and move us a step closer to realizing more of the potential of the LHS vision.

### Future Research

Comprehensive reporting of implementation and evaluation efforts is an important step to moving the LHS field forward. Differences in how implementation determinants and outcomes are reported diminishes the ability to identify trends and important factors across studies and complicates their use in reviews. Increased use of implementation determinant and outcome frameworks will improve the assessment and reporting of barriers, enablers, and implementation outcomes in the field and will improve comparability across studies. However, a word of caution is needed. It would not be desirable for researchers to fall into the trap of being overly focused on what Rapport et al [88] describe as the “theory-drives-change-in-practice” phenomenon, where implementation scientists can be guilty of spending too much time focusing on theories, models, and frameworks, while overlooking the practical and contextual implications of their efforts. We also recognize the need for more rapid implementation science approaches that are flexible and can accommodate rapid-system adaptation. However, at the same time, it is important for a pragmatic approach to be undertaken, in which implementation science frameworks may be used flexibly but pragmatically to guide rapid-cycle design and analysis. As pointed out by Smith et al [89], “striking a balance between rigour, rapidity and flexibility of methods and procedures is difficult” to achieve.

The GRADE level of evidence for empirical LHS studies remains low. Low levels of evidence supporting the value and benefits of an LHS raise complex questions and challenges regarding implementation. Should health care resources be redirected toward implementing new systems whose benefits are not yet empirically proven? Are implementation evaluations the most suitable approach, given LHS research is, by its nature, an applied field of study? In answering these questions, it is important to determine what the right evidence standard is for assessing LHS studies. Medical innovations must typically undergo an evaluation of effectiveness, safety, and cost-effectiveness. If LHSs are intended to directly improve clinical care delivery, then a comparable evidence standard would be required to demonstrate benefits and reassure decision makers regarding potential unintended consequences [90]. Empirical evidence standards for the LHS remain unclear at this stage of the field’s development. It is important for LHSs to demonstrate that the increased investment required to implement infrastructure and systems delivers on its ultimate goal to improve care and patient outcomes, while at the same time not increasing the health care cost burden.

Although several reviews of the LHS literature have emerged in recent years [13-15], there are specific areas that warrant more detailed review in future research. As the number of empirical contributions in the LHS field grows, first, a more in-depth analysis of the specific barriers and enablers identified across studies is needed, with identified barriers and enablers mapped to an implementation determinant framework to enable comparison and identification of trends across studies. Another area ripe for further study is an in-depth review of LHS frameworks and theoretical underpinnings, with an examination of how these frameworks are being applied to support the adoption of LHSs into the health system. Finally, a review showcasing case exemplars in promoting LHSs would be beneficial as empirical contributions continue to flourish.

### Strengths and Limitations

Notable strengths of this review center on our focus on empirical studies and the adoption of an “implementation science” lens. This resulted in a focused review of empirical studies rather than a broader and more theoretical (eg, one that included commentaries and opinion pieces) contribution [13,14]. As a result, our findings identified knowledge gaps and methodological limitations to guide empirical LHS research moving forward. Limitations included the inability to include studies published in languages other than English. Notably, almost three-quarters of the studies were from the United States. Given that the LHS concept was first coined by the US IoM, it is not surprising that many of the studies originate from there. There may be equivalent terms used in other parts of the world, and in other languages other than English, that should be explored in future reviews. We also did not include a gray-literature component, as the aim was to focus on peer-reviewed, high-quality research; however, there is much LHS research identified though a gray-literature search and reference lists in a recent LHS review [15]. We have focused limited attention on the review of service and patient outcomes measured and reported in the included studies, and this warrants further investigation.

### Conclusion

Studies empirically investigating and implementing LHS models have been increasing in recent years. In particular, we are seeing research concerned with implementing a variety of programs, systems, or platforms designed to contribute to achieving an LHS. However, high-quality empirical research, such as randomized controlled trials and implementation evaluations, is still lacking. Comprehensive reporting of implementation and evaluation efforts is an important step in moving the LHS field forward. In particular, the routine use of implementation determinant and outcome frameworks will improve the assessment and reporting of barriers, enablers, and implementation outcomes in this field and will enable comparison and identification of trends across studies. This will enrich our understanding of how to make progress toward an LHS.

## Appendix

Multimedia Appendix 1 Details of included studies.

## Abbreviations

AI: artificial intelligence
CFIR: Consolidated Framework for Implementation Research
DoE: Diffusion of Excellence
ESP: Evidence Synthesis Program
GRADE: Grading of Recommendations Assessment, Development and Evaluation
IoM: Institute of Medicine
LHS: learning health system
MAST: Model for Assessment of Telemedicine
PDSA: plan-do-study-act
PREM: patient-reported experience measure
PRISMA-ScR: Preferred Reporting Items of Systematic Review and Meta-Analyses Extension for Scoping Reviews
PROM: patient-related outcome measure
RAP: rapid assessment procedure
RE-AIM: Reach, Effectiveness, Adoption, Implementation, and Maintenance
VHA: Veterans Health Administration
