# Correction: The Science of Learning Health Systems: Scoping Review of Empirical Research

**DOI:** 10.2196/41424

**Published:** 2022-08-04

**Authors:** Louise A Ellis, Mitchell Sarkies, Kate Churruca, Genevieve Dammery, Isabelle Meulenbroeks, Carolynn L Smith, Chiara Pomare, Zeyad Mahmoud, Yvonne Zurynski, Jeffrey Braithwaite

**Affiliations:** 1 Australian Institute of Health Innovation Macquarie University Sydney Australia

In “The Science of Learning Health Systems: Scoping Review of Empirical Research” (2022;10(2):e34907) the authors noted an error.

In the originally published article, Figure 2 appeared incorrectly (Multimedia Appendix 1). In the corrected version of the article, Figure 2 was updated with the following image:

**Figure 2:**
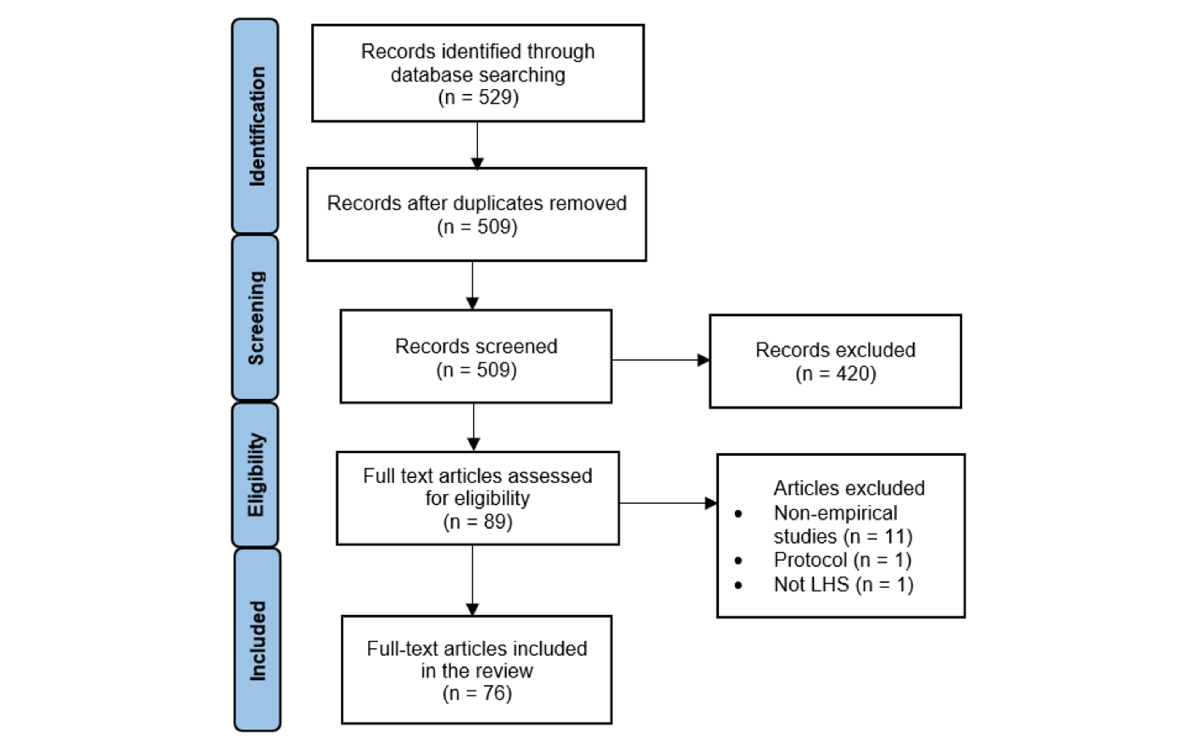
Search and review strategy. LHS: learning health system.

The correction will appear in the online version of the paper on the JMIR Publications website on August 4, 2022, together with the publication of this correction notice. Because this was made after submission to full-text repositories, the corrected article has also been resubmitted to those repositories.

## Appendix

Multimedia Appendix 1 Originally published Figure 1.

