# Supplementary figures and images for: Correction: The Science of Learning Health Systems: Scoping Review of Empirical Research
Source: JMIR Med Inform. 2022 Aug 4;10(8):e41424. doi: 10.2196/41424 (PMC9389390; doi:10.2196/41424)

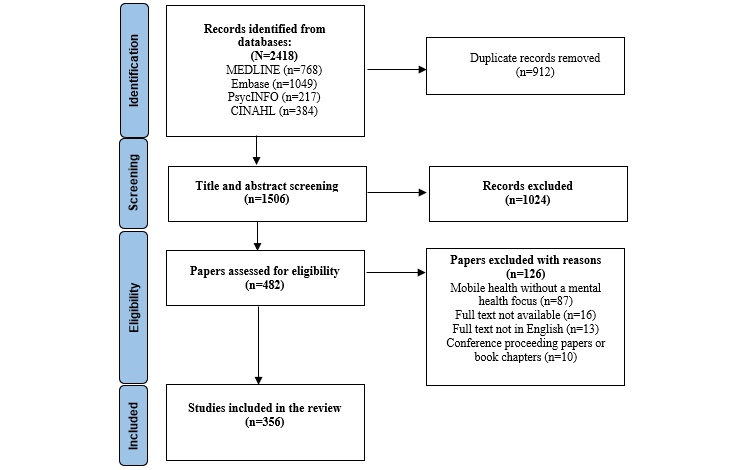

Supplement: Multimedia Appendix 1 [file medinform_v10i8e41424_app1.png]
